# Hypercholesterolemia Correlates With Glomerular Phospholipase A2 Receptor Deposit and Serum Anti-Phospholipase A2 Receptor Antibody and Predicts Proteinuria Outcome in Idiopathic Membranous Nephropathy

**DOI:** 10.3389/fimmu.2022.905930

**Published:** 2022-06-17

**Authors:** Lei Dong, Yue-qiang Li, Shui-ming Guo, Gang Xu, Wang Wei, Min Han

**Affiliations:** ^1^ Department of Nephrology, Tongji Hospital, Tongji Medical College, Huazhong University of Science and Technology, Wuhan, China; ^2^ Department of Gastroenterology, Tongji Hospital, Tongji Medical College, Huazhong University of Science and Technology, Wuhan, China

**Keywords:** hyperlipidemia, phospholipase A2 receptor, membranous nephropathy, proteinuria, hypercholesterolemia

## Abstract

**Background:**

The anti-phospholipase A2 receptor (PLA2R) antibody is a non-invasive diagnostic tool and prognosis predictor of idiopathic membranous nephropathy (IMN). Baseline hypercholesterolemia independently predicts proteinuria outcomes in IMN patients. Thus, we investigated whether hyperlipidemia is correlated with anti-PLA2R and pathological indicators.

**Methods:**

A total of 495 IMN patients identified by kidney biopsy in Wuhan Tongji Hospital, China, from January 2016 through December 2020 were enrolled in this study. Data on clinical features, pathology findings, and outcomes were collected.

**Results:**

Total cholesterol (TC), non-high-density lipoprotein cholesterol (non-HDL-C), low-density lipoprotein cholesterol (LDL-C), and triglyceride (TG) were positively related to proteinuria, indicating damage to the renal glomerulus [Spearman’s rank correlation coefficient = 0.432, 0.462, 0.315, and 0.289, respectively, *P* < 0.001 for all]. In univariate logistic regression, low HDL-C [odds ratio (OR): 0.856; 95% CI: 0.778–0.939; *P* = 0.001] and high TG [OR: 1.025; 95% CI: 1.006–1.044; *P* = 0.011] were correlated with tubular atrophy, suggesting lesions on tubules. Increased TC [adjusted OR: 1.285; 95% CI: 1.119–1.475; *P* < 0.001], non-HDL-C [adjusted OR: 1.284; 95% CI: 1.113–1.482; *P* = 0.001], and LDL-C [adjusted OR: 1.178; 95% CI: 1.009–1.376; *P* = 0.039] independently predicted glomerular PLA2R deposit; similar results were observed for lipids in predicting the seropositivity of anti-PLA2R antibodies. After treatment, increased HDL-C [adjusted hazard ratio (HR): 1.764; 95% CI: 1.241–2.507; *P* = 0.002] and decreased non-HDL-C [adjusted HR: 0.884; 95% CI: 0.795–0.983; *P* = 0.022] independently predicted proteinuria remission.

**Conclusion:**

Hypercholesterolemia is a potentially useful biomarker for disease severity, serum anti-PLA2R antibody, glomerular PLA2R deposit, and proteinuria outcome of IMN.

## Introduction

Idiopathic membranous nephropathy (IMN) is a common glomerulonephritis disorder pathologically characterized by an apparent thickening of the capillary walls due to immune complex deposits (1). The immune complexes comprise immunoglobulin G (IgG), the recently recognized relevant antigens, such as phospholipase A2 receptor (PLA2R) and thrombospondin domain-containing 7A (THSD7A), and complement components (2). Anti-PLA2R antibody, the major autoantibody of podocytes, was detectable in about 70%–80% of patients with membranous nephropathy (MN) when diagnosed (3). The seropositivity of the anti-PLA2R antibody can be detected from months to years before the clinical diagnosis of MN (4). Due to its high specificity, the anti-PLA2R antibody has been considered a non-invasive tool for the diagnosis of MN (5). Furthermore, the decline in serum antibody titers predicted a decrease in proteinuria over several months in patients with MN (6, 7). However, the seropositivity of the anti-PLA2R antibody is not always concurrent to glomerular anti-PLA2R binding (3, 8). In some MN patients with positive glomerular PLA2R staining, circulating anti-PLA2R antibodies may be absent at admission but become detectable during follow-up or relapse (9). Thus, it is speculated that circulating anti-PLA2R antibodies are prevalent only when they are saturated in kidney tissues (8). Although several studies have been focused on PLA2R in the last decade, the mechanism underlying its role in pathogenesis remains to be elucidated.

Hyperlipidemia is common in IMN patients, especially in the case of nephrotic syndrome. However, the lack of knowledge about its role in the pathogenesis and outcome leads it to be out of the spotlight. Clinical parameters including declined glomerular filtration rate, massive proteinuria, and low serum albumin level are known risk factors for progression of kidney function in MN (10), while lipids are not mentioned. In the present study, triglyceride (TG), total cholesterol (TC), high-density lipoprotein cholesterol (HDL-C), low-density lipoprotein cholesterol (LDL-C), and non-high-density lipoprotein cholesterol (non-HDL-C) were evaluated as lipid profiles and their roles evaluated in IMN. In a previous study, we demonstrated that hypercholesterolemia at baseline is an independent risk factor for non-remission of proteinuria in IMN patients (11). However, whether hyperlipidemia is correlated with the outcomes *via* any pathological parameter remains to be clarified. Thus, we examined the correlations between lipids and both pathological parameters and circulating anti-PLA2R antibody titers in the “real world” and further characterized the effect of lipid control on the outcome of proteinuria.

## Materials and Method

### Study Design and Patients

This retrospective, single-center, cohort study included IMN patients proven by native kidney biopsy from January 1, 2016, to December 31, 2020, from Tongji Hospital in Wuhan, China. The eligible participants were ≥18 years old with biopsy-proven IMN. Patients with the following criteria were excluded: (i) use of steroid and immunosuppressants within 6 months before screening; (ii) use of lipid-lowering medication upon admission; and (iii) MN secondary to known causes, including hepatitis virus infection, tumor, autoimmune disease, and metabolic disease. Data extraction and analyses were conducted as described previously (11).

This study was in accordance with the Helsinki declaration. The Ethics Review Board of Tongji Hospital, Tongji Medical College, Huazhong University of Science and Technology, waived the requirement of informed consent due to the retrospective design of the study (No. TJ-IRB20210701).

### Data Collection, Therapy, and Definitions

**Figure 1:**
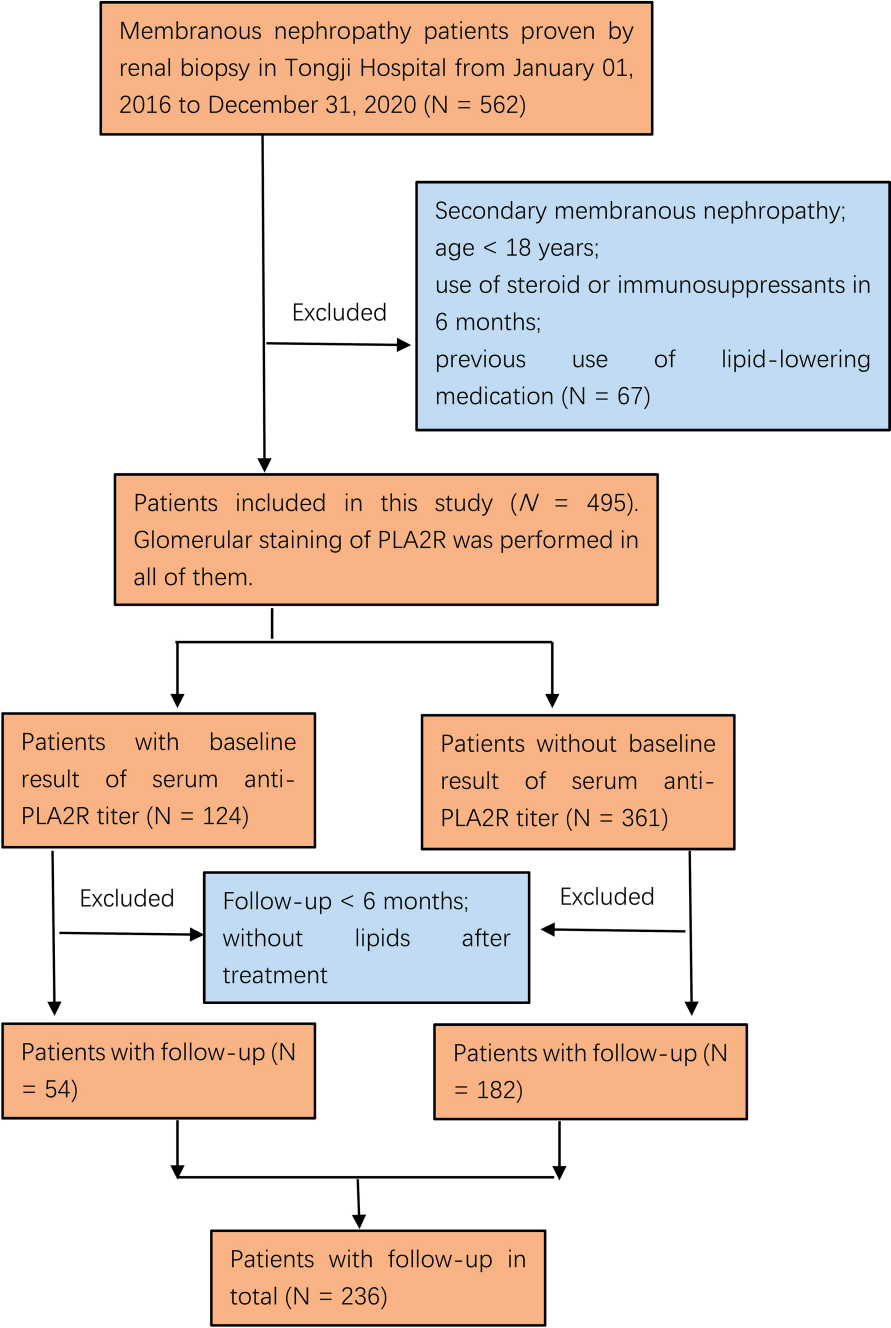
Flowchart of participant selection in this study. Among 562 membranous nephropathy patients, 495 who met the inclusion criteria were included. One hundred twenty-four of 495 participants had been examined for the serum anti-PLA2R antibody at onset, and 236/495 had longitudinal data with follow-up for >6 months.

Demographic, anthropometric, clinical, biochemical, and pathological parameters were retrieved from the electronic medical records of Tongji Hospital. A total of 495 patients with IMN were included, and 236 had consecutive data for a follow-up of >6 months. Data of glomerular staining for PLA2R were available in all of the 495 patients, while circulating anti-PLA2R antibody titers on admission were acquired for only 124 patients (**Figure 1**).

**Figure 2:**
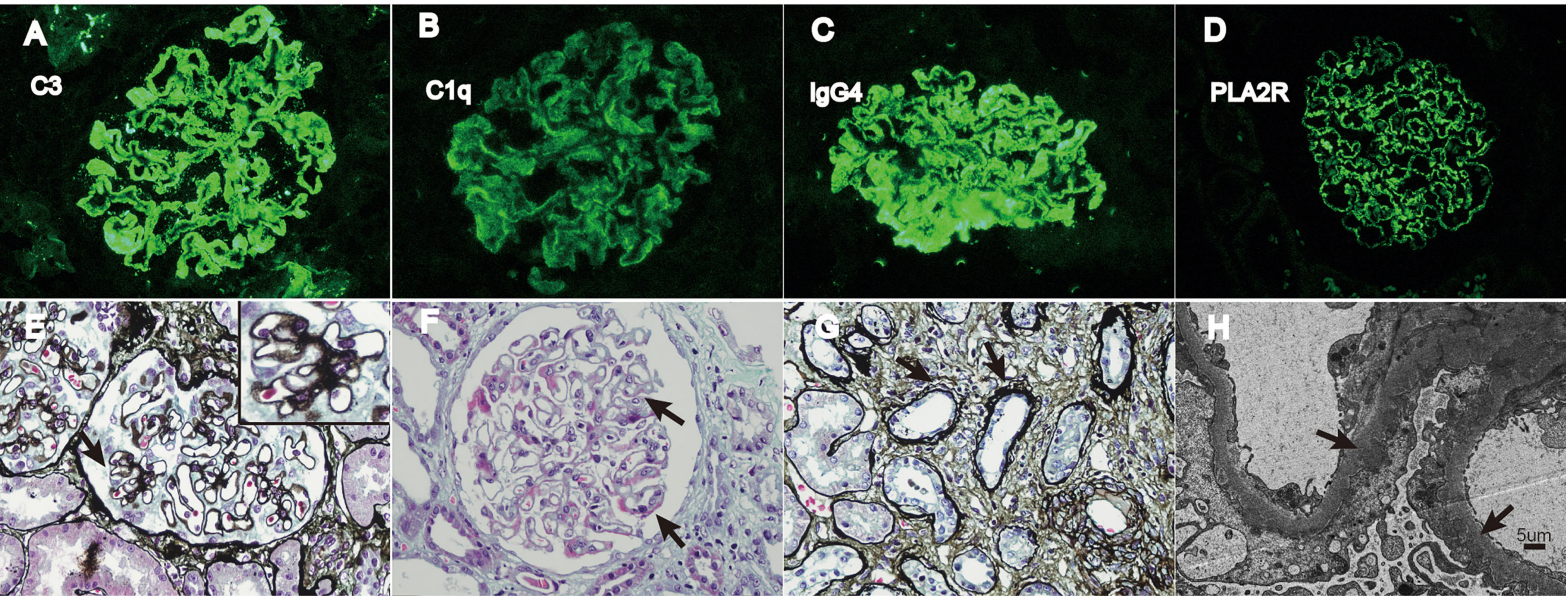
Typical pathological images of IMN. **(A–D)** Immunofluorescence of C3, C1q, IgG4, and PLA2R deposit, respectively (×400). **(E)** The black arrow indicates spike-like projections in glomerulus (PASM stain, ×400). **(F)** The black arrow indicates immune complex deposit under epithelial cells (Masson stain, ×400). **(G)** The black arrow shows atrophy renal tubules (PASM stain, ×400). **(H)** The black arrow shows dense deposit under epithelial cells (SEM, ×3000). PASM = periodic acid-silver methenamine and Masson stain; SEM = scanning electron microscope.

The baseline biochemical indicators, including albumin, serum creatinine, urine protein, and lipid profiles, were measured during the first hospitalization before kidney biopsy. All lipid parameters were assessed in the non-fasting specimens and measured by enzyme colorimetry. Indirect immunofluorescent assay was utilized to detect PLA2R deposit in kidney tissue (**Supplementary Figure 1**) and the qualitative determination of the serum anti-PLA2R antibody. Enzyme-linked immunosorbent assay was conducted for the quantitative determination of the serum anti-PLA2R antibody. The pathology reports were reviewed to assess the following features: IgG4-dominant deposit (**Supplementary Figure 1**) defined as the immunofluorescence intensity of IgG4 higher than or equal to any other subgroups (12); IgA, IgM, C3, C4, C1q, and PLA2R deposit; Ehrenreich–Churg morphological stage; occurrence of diffuse mesangial proliferation and crescent formation; and tubular (T) atrophy graded as T0 (absent), T1 (<25%), T2 (25%–50%), and T3 (>50%). The typical images are shown in **Figure 2**.

The supportive and immunosuppressive therapy was at the discretion of the nephrologists according to the disease severity of the patients. Angiotensin-converting enzyme inhibitors (ACEis) or angiotensin II receptor blockers (ARBs) were utilized as supportive treatment at the maximally tolerated dose (13). A statin treatment, also a supportive treatment, was started in patients with nephrotic syndrome and dyslipidemia (13). Immunosuppressive therapy included corticosteroids, cyclophosphamide, calcineurin inhibitors, mycophenolate mofetil, and rituximab (14). Based on Kidney Disease: Improving Global Outcomes (KDIGO) guidelines, complete remission was defined as proteinuria <0.5 g/day, and partial remission was defined as proteinuria <3.5 g/day, which concurrently achieved a ≥50% reduction compared to the baseline; in both cases, the kidney function was stable (14). In this study, complete and partial remission were considered remission since both served as surrogate endpoints in MN (15).

### Statistical Analysis

Continuous variables were tested for normality and were found to be non-normally distributed. Skew variables were expressed as median with interquartile range (IQR), and categorical variables were presented as count and percentage. The bivariate and multinomial logistic regression analyses were performed to evaluate the association between lipid profiles and pathological kidney parameters. Multivariable logistic regression models were utilized to evaluate the independent association of lipids and PLA2R deposit and IgG4-dominant deposit in kidneys. The bivariate association between lipids and serum anti-PLA2R titers was evaluated by Spearman analysis. Furthermore, the independent correlations of these two variables were tested using multivariable logistic regression models. The predictive abilities of lipid improvement in proteinuria remission were assessed by Cox regression analysis. All statistical tests were performed using software SPSS (version 24.0, IBM, USA). *P*-value < 0.05 indicated statistically significant difference.

## Result

### Baseline Characteristics

A total of 495 participants were included in this study, and their baseline characteristics are presented in **Table 1**. The median age of the cohort was 49 (IQR 39–56) years, and median baseline serum creatinine was in the normal range. The median urine protein was in the nephrotic range (>3.5 g/day), and that of serum albumin was <30 g/l. The immunohistological staining showed that glomerular C3, PLA2R, and IgG4-dominant deposits were most common, which is consistent with the features of IMN. The proportion of the glomerular PLA2R deposit was 81.8%, similar to that described previously (1). At baseline, patients were mostly at the early morphological stages (I + II, 89.1%) and had mild tubular atrophy (74.7%). About 64.8% of patients received immunosuppressive therapy during follow-up.

**Table 1:** Baseline characteristics of idiopathic membranous nephropathy patients.

All patients (*N* = 495)			
Demographics		Pathological parameters, no. (%)	
Age, years	49 (39–56)	IgG4 dominant deposit	336 (67.8)
Male sex, no. (%)	282 (58.1)	IgA deposit	79 (15.9)
**Ethnicity**		IgM deposit	131 (26.4)
Asian, no. (%)	495 (100)	C3 deposit	425 (85.8)
**Anthropometric measurements**		C4 deposit	99 (20.0)
Systolic BP, mm Hg	128 (120–136)	C1q deposit	126 (25.4)
Diastolic BP, mm Hg	82 (76–90)	PLA_2_R deposit	405 (81.8)
BMI, kg/m^2^	24.1 (22.2–26.1)	Morphological staging	
**Kidney function measurements**		I+II	441 (89.1)
Serum creatinine, μmol/L	75 (60–90)	III+IV	54 (10.9)
Urine protein, g/day	3.79 (1.84–6.09)	Mesangial proliferation	37 (7.4)
Albumin, g/L	28.8 (23.5–34.0)	Lesions of FSGS	54 (10.9)
**Plasma lipid levels**		Crescent formation	40 (8.1)
TC, mmol/L	6.37 (5.12–7.90)	Tubular atrophy	
Non-HDL-C, mmol/L	4.93 (3.84–6.58)	T0	42 (8.5)
HDL-C, mmol/L	1.27 (1.03–1.61)	T1	370 (74.7)
LDL-C, mmol/L	3.72 (2.81–4.99)	T2	82 (16.6)
Triglyceride, mmol/L	2.28 (1.44–3.66)	T3	1 (0.2)
**Treatment**			
Immunosuppressive therapy, no. (%)	321 (64.8%)		

T, tubular.

### Lipid Profile Is Correlated With the Severity of IMN

**Figure 3:**
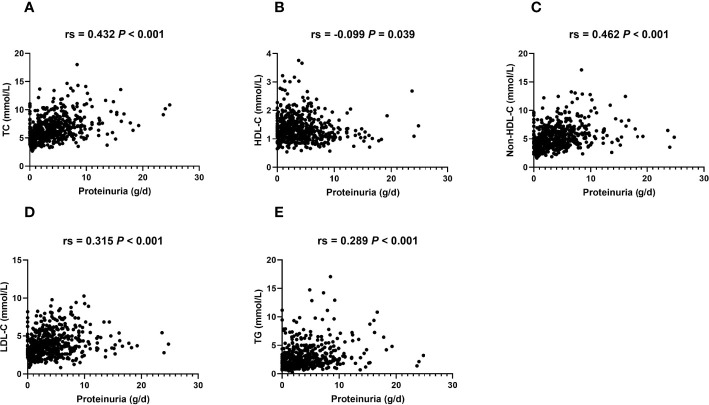
Serum lipid levels are related to proteinuria in IMN patients. Bivariate scatterplots relating proteinuria to TC **(A)**, HDL-C **(B)**, non-HDL-C **(C)**, LDL-C **(D)**, and TG **(E)**, including *P-*values for Spearman’s analysis (N = 495). Rs: Spearman’s rank correlation coefficient.

Proteinuria is concurrent with the damage to renal glomerulus. **Figure 3** shows that TC, non-HDL-C, LDL-C, and TG at baseline were positively related to proteinuria, while HDL-C exhibited an inverse correlation with proteinuria, indicating a correlation between lipids and glomerular lesions. Moreover, damage on tubules presenting as tubular atrophy correlating with lipids is shown in **Table 2**. Decreased HDL-C and elevated TG were related to severe tubular atrophy. These data suggested that lipids were correlated with renal glomerulus and tubule damage.

**Table 2:** Logistic regression analyses for lipid profiles and pathological indicators except for IgG4 dominant and PLA2R deposit.

	TC (N = 495)		HDL (N = 495)		Non-HDL (N = 495)		LDL (N = 495)		TG (N = 495)	
	OR (95% CI)	*P*	OR (95% CI)	*P*	OR (95% CI)	*P*	OR (95% CI)	*P*	OR (95% CI)	*P*
IgA	0.917 (0.815–1.031)	0.145	0.756 (0.431–1.325)	0.328	0.926 (0.818–1.047)	0.219	0.893 (0.765–1.043)	0.154	1.056 (0.957–1.166)	0.276
C3	1.085 (0.961–1.226)	1.189	1.157 (0.663–2.017)	0.608	1.082 (0.953–1.227)	0.224	1.137 (0.969–1.335)	0.116	0.981 (0.882–1.092)	0.729
C4	0.999 (0.905–1.103)	0.988	0.962 (0.599–1.544)	0.871	1.007 (0.909–1.114)	0.897	1.030 (0.906–1.171)	0.648	0.970 (0.877–1.073)	0.557
C1q	0.918 (0.833–1.012)	0.085	1.161 (0.757–1.779)	0.494	0.902 (0.813–1.000)	0.050^*^	0.947 (0.837–1.071)	0.387	0.875 (0.784–0.977)	0.018^*^
Morphological staging	1.012 (0.989–1.036)	0.299	0.927 (0.826–1.040)	0.198	1.023 (0.999–1.049)	0.065	1.019 (0.987–1.052)	0.239	1.001 (0.977–1.024)	0.961
Mesangial proliferation	0.891 (0.751–1.058)	0.188	0.490 (0.193–1.246)	0.134	0.923 (0.769–1.108)	0.392	0.924 (0.739–1.155)	0.487	1.027 (0.889–1.186)	0.717
Crescent formation	1.080 (0.945–1.236)	0.259	0.685 (0.319–1.470)	0.331	1.118 (0.978–1.277)	0.101	1.167 (0.979–1.391)	0.084	1.000 (0.868–1.151)	0.997
Tubular atrophy	1.011 (0.991–1.031)	0.264	0.856 (0.778–0.939)	0.001^**^	1.020 (1.000–1.042)	0.055	1.004 (0.978–1.030)	0.759	1.025 (1.006–1.044)	0.011^*^

OR, odds ratio; CI, confidence interval.

*P < 0.05; **P < 0.01.

The correlations between lipids and other pathological parameters are summarized in **Table 2**. No significant correlations were noted in lipid profiles with respect to glomerular IgA, C3, C4 deposit, morphological staging, mesangial proliferation, and crescent formation (**Table 2**).

C1q deposit indicated the classical pathway of complement activation. Decreased non-HDL-C and TG were related to C1q deposit (**Table 2**), suggesting a putative role of lipids in the activation of the complement system.

### Lipids Correlated With Glomerular PLA2R Deposit and Anti-PLA2R Antibody

Notably, lipid profiles were significantly relevant to the characteristic features of IMN, including glomerular IgG4-dominant and PLA2R deposits (**Table 3** and **Table 4**). The baseline characteristics are listed in **Supplementary Tables 1 and 2**. Increased TC and non-HDL-C were implied as independent risk factors for IgG4-dominant staining after adjusting for gender, age, body mass index (BMI), blood pressure (BP), serum creatinine (SCr), and albumin (**Table 3**). Simultaneously, elevated TC, non-HDL-C, and LDL-C were independently associated with glomerular PLA2R deposit after adjustment of gender, age, BMI, BP, SCr, and albumin (**Table 4**). Increased TG was a risk factor for positive PLA2R staining but turned out to be insignificant after further adjustment (**Table 4**).

**Table 3:** Logistic regression analyses for lipid profiles and IgG4 dominant deposit.

	Model 1 (N = 495)		Model 2 (N = 495)		Model 3 (N = 495)	
	OR (95% CI)	*P*	Adj. OR (95% CI)	*P*	Adj. OR (95% CI)	*P*
TC	1.107 (1.020–1.202)	0.015^*^	1.102 (1.005–1.209)	0.040^*^	1.098 (1.011–1.193)	0.027^*^
HDL-C	1.105 (0.749–1.629)	0.616	–	–	–	–
Non-HDL-C	1.097 (1.008–1.196)	0.033^*^	1.112 (1.014–1.220)	0.025^*^	1.095 (1.006–1.192)	0.037^*^
LDL-C	1.088 (0.976–1.213)	0.126	–	–	–	–
TG	1.068 (0.987–1.156)	0.101	–	–	–	–

Model 1 was univariate analysis; model 2 was adjusted for gender, age, BMI, BP, and SCr; model 3 was adjusted for all the variables in model 2 plus serum albumin.

OR, odds ratio; CI, confidence interval; Adj., adjusted; BMI, body mass index; BP, blood pressure; SCr, serum creatinine.

*P< 0.05.

**Table 4:** Logistic regression analyses for lipid profiles and PLA2R deposit.

	Model 1 (N = 495)		Model 2 (N = 495)		Model 3 (N = 495)	
	OR (95% CI)	*P*	Adj. OR (95% CI)	*P*	Adj. OR (95% CI)	*P*
TC	1.348 (1.177–1.544)	<0.001^**^	1.267 (1.090–1.474)	0.002^**^	1.285 (1.119–1.475)	<0.001^**^
HDL-C	1.255 (0.742–2.124)	0.397	–	–	–	–
Non-HDL-C	1.382 (1.191–1.604)	<0.001^**^	1.289 (1.092–1.520)	0.003^**^	1.284 (1.113–1.482)	0.001^**^
LDL-C	1.245 (1.063–1.459)	0.007^**^	1.209 (1.011–1.446)	0.038^*^	1.178 (1.009–1.376)	0.039^*^
TG	1.175 (1.028–1.343)	0.018^*^	1.107 (0.953–1.286)	0.182	–	–

Model 1 was univariate analysis; model 2 was adjusted for gender, age, BMI, BP, and SCr; model 3 was adjusted for all the variables in model 2 plus serum albumin.

OR, odds ratio; CI, confidence interval; Adj., adjusted; BMI, body mass index; BP, blood pressure; SCr, serum creatinine.

*P < 0.05; **P < 0.01.

**Figure 4:**
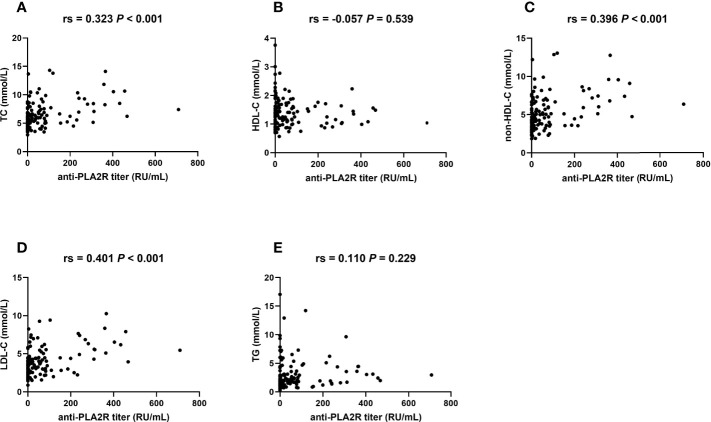
TC, non-HDL-C, and LDL-C are correlated with serum anti-PLA2R titers. Bivariate scatterplots relating anti-PLA2R titers to TC **(A)**, HDL-C **(B)**, non-HDL-C **(C)**, LDL-C **(D)**, and TG **(E)**, including *P*-values for Spearman’s analysis (N = 124). Rs: Spearman’s rank correlation coefficient.

Since glomerular PLA2R staining is not always parallel to seropositivity anti-PLA2R antibody, we further studied the association between lipid parameters and serum anti-PLA2R antibody in 124 patients available for serum anti-PLA2R titer on admission. The baseline characteristics of this population are summarized in **Supplementary Table 3**. The correlations between lipids and seropositivity of the anti-PLA2R antibody were analyzed using a multivariate logistic regression model, and the correlations between lipids and serum antibody titer were analyzed by Spearman’s analysis. Elevated TC, non-HDL-C, and LDL-C levels were independent risk factors for seropositivity of the anti-PLA2R antibody (**Table 5**). Also, significant positive correlations were established between TC, non-HDL-C, LDL-C levels, and serum anti-PLA2R titers (**Figure 4**).

**Table 5:** Logistic regression analyses for lipid profiles and seropositivity of the anti-PLA2R antibody.

	Model 1 (N = 124)		Model 2 (N = 124)		Model 3 (N = 124)	
	OR (95% CI)	*P*	Adj. OR (95% CI)	*P*	Adj. OR (95% CI)	*P*
TC	1.258 (1.054–1.502)	0.011^*^	1.232 (1.031–1.472)	0.022^*^	1.262 (1.057–1.508)	0.010^*^
HDL-C	0.684 (0.335–1.396)	0.297	–	–	–	–
Non-HDL-C	1.296 (1.077–1.558)	0.006^**^	1.256 (1.042–1.514)	0.017^*^	1.302 (1.082–1.568)	0.005^**^
LDL-C	1.471 (1.158–1.868)	0.002^**^	1.440 (1.134–1.829)	0.003^**^	1.484 (1.168–1.886)	0.001^**^
TG	0.971 (0.852–1.107)	0.66	–	–	–	–

Model 1 was univariate analysis; model 2 was adjusted for gender, age, BMI, BP, and SCr; model 3 was adjusted for all the variables in model 2 plus serum albumin.

OR, odds ratio; CI, confidence interval; Adj., adjusted; BMI, body mass index; BP, blood pressure; SCr, serum creatinine.

*P < 0.05; **P < 0.01.

**Figure 5:**
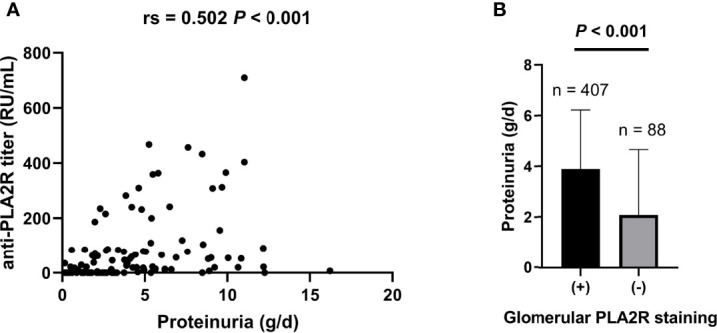
Both baseline serum anti-PLA2R titer and glomerular PLA2R deposits are correlated with proteinuria. **(A)** Spearman’s analysis was applied for correlations between baseline serum anti-PLA2R titer and proteinuria (N = 124). **(B)** Mann–Whitney U test was applied to analyze the correlations between tissue PLA2R deposit and proteinuria (N = 495). Rs: Spearman’s rank correlation coefficient.

Similar to the previous study (16), both anti-PLA2R titer and glomerular PLA2R staining were correlated with proteinuria (**Figure 5**), confirming the clinical significance of the anti-PLA2R antibody and tissue PLA2R deposit.

### Lipids Predicted Proteinuria Remission Post-Therapy

Previously, we reported that low TC and non-HDL-C levels pre-therapy were independent predictors for proteinuria remission in IMN patients (11). To determine the effect of lipid control on proteinuria remission, a multivariate Cox regression analysis was carried out on 236 participants who had complete follow-up data. The characteristics of these participants are listed in **Supplementary Table 4**. Increased HDL-C and decreased non-HDL-C levels post-therapy independently predicted proteinuria remission after adjustment of age, gender, BMI, BP, SCr, and albumin (**Table 6**). Conversely, decreased LDL-C predicted proteinuria remission after treatment but became insignificant after adjustment of albumin (**Table 6**).

**Table 6:** Cox proportional hazard models for the associations between lipids after treatment and proteinuria remission.

	Model 1 (N = 236)		Model 2 (N = 236)		Model 3 (N = 236)	
	HR (95% CI)	*P*	Adj. HR (95% CI)	*P*	Adj. HR (95% CI)	*P*
TC	0.912 (0.832–1.000)	0.051	–	–	–	–
HDL-C	1.659 (1.167–2.359)	0.005^**^	1.675 (1.182–2.375)	0.004^**^	1.764 (1.241–2.507)	0.002^**^
Non-HDL-C	0.858 (0.771–0.954)	0.005^**^	0.860 (0.774–0.956)	0.005^**^	0.884 (0.795–0.983)	0.022^*^
LDL-C	0.878 (0.776–0.993)	0.038^*^	0.872 (0.770–0.988)	0.032^*^	0.870 (0.768–1.012)	0.107
TG	0.957 (0.844–1.086)	0.499	–	–	–	–

Model 1 was univariate analysis; model 2 was adjusted for gender, age, BMI, BP, and SCr; model 3 was adjusted for all the variables in model 2 plus serum albumin.

HR, hazard ratio; CI, confidence interval; Adj., adjusted; BMI, body mass index; BP, blood pressure; SCr, serum creatinine.

*P < 0.05; **P < 0.01.

## Discussion

PLA2R has been considered the major target antigen for IMN, and anti-PLA2R is a widely accepted diagnostic and prognostic biomarker (17). Our preliminary results indicated that hypercholesterolemia at baseline is an independent risk factor for persistent proteinuria in IMN (11). Herein, we found that hypercholesterolemia at onset was correlated with glomerular and tubular lesions, glomerular PLA2R deposit, and serum anti-PLA2R titers in a large Chinese cohort. Also, positive effects on proteinuria remission by lipid control were observed in the overall population. These data demonstrated that dyslipidemia is a risk factor not only for anti-PLA2R but also for non-remission of proteinuria and may be a potential therapeutic target in IMN.

The current data indicated a link between TG, HDL-C, and damage to renal tubules. In addition, renal TG accumulation was accompanied by interstitial fibrosis in the tubulointerstitium in a diabetic nephropathy rat model (18). Pravastatin attenuated tubulointerstitial fibrosis in the rat model of cyclosporine-induced nephropathy (19). These results might imply a critical role of lipid control in the attenuation of tubulointerstitial lesions in MN.

The pathology of IMN is driven by the activation of the complement system, inducing podocyte injury and proteinuria (20). Reportedly, all the three pathways of complement activation, the classical, the alternative, and the lectin pathways, are active in IMN, although at various intensities (21). In this cohort, a majority (85.8%) of the participants had positive staining of C3, and a few (25.4%) were positive for the C1q deposit, in accordance with previous findings (21). IgG4 is a predominant subclass in the subepithelial immune complexes and has been speculated to activate the lectin pathway and induce podocyte injury in anti-PLA2R-associated MN (22, 23). In the present study, increased TC was an independent risk factor for the IgG4 deposit, demonstrating a potential role of hypercholesterolemia in the pathogenesis of MN. However, IgG4 could not produce complement activation through the classical pathway (22). How the classical pathway was initiated is yet to be elucidated. Decreased non-HDL-C and TG were potentially related to the C1q deposit, providing us a new perspective for further studies.

The present study indicated that increased TC, non-HDL-C, and LDL-C were independent risk factors for the glomerular PLA2R deposit. In addition, high TC, non-HDL-C, and LDL-C independently predicted the seropositivity of anti-PLA2R. Therefore, our data revealed hypercholesterolemia as a valuable biochemical predictor for the glomerular PLA2R deposit and sero-anti-PLA2R titer. Consistent with our data, a meta-analysis, including 20 studies involving 2,224 IMN patients, showed that TC was correlated with serum anti-PLA2R and glomerular PLA2R deposit (24). However, the remaining lipid profiles were not included in the meta-analysis. Despite this strong association, it is unclear how hyperlipidemia is correlated with PLA2R in MN.

To date, only a few studies have focused on the role of hyperlipidemia in the outcomes of glomerulonephropathies. In a multicenter cohort, including 761 children with primary glomerular diseases, patients not achieving remission exhibited a significantly high rate of hypercholesterolemia than those in remission (25). Hypertriglyceridemia predicts poor renal survival in an IgA cohort (26). In a previous study, we found that low TC and non-HDL-C levels at onset predicted a high remission of proteinuria; however, whether a decrease in lipids is beneficial is yet to be elucidated. The current data suggested that non-HDL-C post-therapy is correlated with proteinuria outcome in IMN. LDL-C post-therapy has a similar trend to be correlated with proteinuria outcome, although not independently. Supporting our findings, Rayner et al. found that simvastatin reduced proteinuria and increased serum albumin levels in a small prospective study clinical trial (27). Sprangers et al. reported that use of statins was an independent protective factor for renal outcome in a retrospective study with a long-term follow-up (28). Thereby, additional studies focused on the target levels of lipids are needed and whether enhanced lipid-lowering therapy is essential remains to be elucidated.

To the best of our knowledge, this is the first study to address the correlations between lipid profiles and anti-PLA2R. The data relied on high-quality records from an academic center with long-term follow-up. The limitations of this study were as follows: retrospective design; lack of data from other centers; absence of systematic monitoring for anti-PLA2R titers; and a small number of patients with longitudinal data of lipids during follow-up. Thus, additional studies with multicenter data focusing on the correlations between consecutive changes in lipids and anti-PLA2R titers are needed. Currently, a large gap remains in the understanding of the correlation between hypercholesterolemia and anti-PLA2R, emphasizing the need to explore the underlying mechanisms.

In conclusion, we demonstrated that baseline hypercholesterolemia is an independent risk factor for the PLA2R deposit in kidney tissues and also for seropositivity of anti-PLA2R. Hypercholesterolemia also predicted persistent proteinuria in IMN patients. These findings might form a basis for understanding the pathogenesis of IMN.

## Data Availability Statement

The raw data supporting the conclusions of this article will be made available by the authors, without undue reservation.

## Ethics Statement

The studies involving human participants were reviewed and approved by the ethical review board of Tongji Hospital, Tongji Medical College, Huazhong University of Science and Technology. Written informed consent for participation was not required for this study in accordance with the national legislation and the institutional requirements.

## Author Contributions

LD was the main investigator, collected the clinical and demographic data of the patients, analyzed the data, and prepared the manuscript. Y-qL and S-mG reviewed the pathology reports. GX provided the conception and design and revised the manuscript. WW reviewed the clinical data, analyzed the data, and prepared and finalized the manuscript. MH initiated the report, was the main supervisor during all stages, and revised and finalized the manuscript for publication. All authors contributed to the article and approved the submitted version.

## Funding

This study was supported by a grant from the National Natural Science Foundation of China (No. 81800654).

## Conflict of Interest

The authors declare that the research was conducted in the absence of any commercial or financial relationships that could be construed as a potential conflict of interest.

## Publisher’s Note

All claims expressed in this article are solely those of the authors and do not necessarily represent those of their affiliated organizations, or those of the publisher, the editors and the reviewers. Any product that may be evaluated in this article, or claim that may be made by its manufacturer, is not guaranteed or endorsed by the publisher.

## Abbreviations

IMN, idiopathic membranous nephropathy; IgG, immunoglobulin G; PLA2R, phospholipase A2 receptor; THSD7A, thrombospondin domain-containing 7A; MN, membranous nephropathy; TG, triglyceride; TC, total cholesterol; HDL-C, high-density lipoprotein cholesterol; LDL-C, low-density lipoprotein cholesterol; non-HDL-C, non-high-density lipoprotein cholesterol; T, tubular; ACEi, angiotensin-converting enzyme inhibitors; ARBs, angiotensin II receptor blockers; KDIGO, Kidney Disease: Improving Global Outcomes; IQR, interquartile ranges; BMI, body mass index; BP, blood pressure; SCr, serum creatinine; sPLA2 IB, group IB secretory phospholipase A2.
