# Supplementary material for: Hypercholesterolemia Correlates With Glomerular Phospholipase A2 Receptor Deposit and Serum Anti-Phospholipase A2 Receptor Antibody and Predicts Proteinuria Outcome in Idiopathic Membranous Nephropathy
Source: Front Immunol. 2022 Jun 17;13:905930. doi: 10.3389/fimmu.2022.905930 (PMC9248763; doi:10.3389/fimmu.2022.905930)
Supplement: Supplementary file 1 [file DataSheet_1.docx]

Supplementary Material

# Supplementary Data


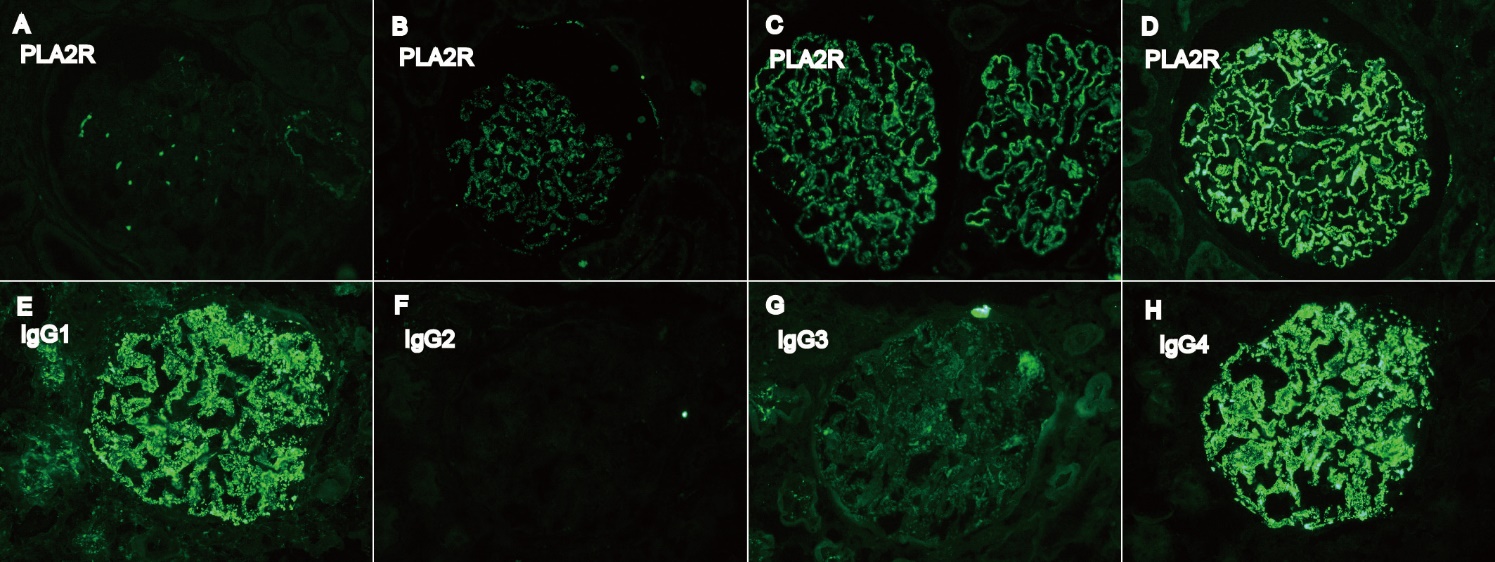


Supple. Figure 1. Typical immunofluorescent images of glomerular PLA2R deposit and IgG4 dominant deposit. (A-D) Immunofluorescence staining intensity of 0, 1+, 2+ and 3+ for PLA2R deposit, respectively (×400). Grades from 1+ to 3+ are defined as positive staining. (E-H) Immunofluorescence staining of IgG1, IgG2, IgG3 and IgG4 for 2+, 0, 1+ and 3+, respectively, from the same patient (×400).

Supple. Table 1. Baseline characteristics of 495 patients according to glomerular IgG4 deposit.

| Characteristics | All patients  (N=495) | IgG4 deposit  (+) (N=336) | IgG4 deposit  (-) (N=159) | *P*-value |
| --- | --- | --- | --- | --- |
| Demographics |  |  |  |  |
| Age, y | 49 (39-56) | 51 (42-55) | 48 (38-56) | 0.323 |
| Male sex, no. (%) | 282 (56.9) | 184 (54.7) | 98 (61.6) | 0.738 |
| Anthropometric Measurements |  |  |  |  |
| Systolic BP, mmHg | 127 (118-139) | 127 (118-138) | 127 (117-139) | 0.962 |
| Diastolic BP, mmHg | 82 (75-91) | 81 (75-90) | 82 (74-91) | 0.744 |
| BMI, kg/m^2^ | 24.1 (22.2-26.1) | 24.6 (22.7-26.9) | 23.6 (21.6-26.2) | 0.008**^**^** |
| Kidney Function Measurements |  |  |  |  |
| Serum creatinine, μmol/L | 75 (60-90) | 73 (60-90) | 76 (61-91) | 0.508 |
| Urine protein, g/d | 3.8 (1.8-6.1) | 3.9 (2.0-6.0) | 3.6 (1.7-6.2) | 0.356 |
| Albumin, g/L | 28.8 (23.5-34.0) | 27.9 (23.1-32.3) | 28.9 (23.6-35.1) | 0.136 |
| Plasma Lipid Levels |  |  |  |  |
| TC, mmol/L | 6.38 (5.14-7.88) | 6.73 (5.36-8.26) | 6.14 (5.00-7.69) | 0.008**^**^** |
| Non-HDL-C, mmol/L | 4.96 (3.85-6.51) | 5.13 (4.08-6.83) | 4.85 (3.63-6.21) | 0.010**^*^** |
| HDL-C, mmol/L | 1.27 (1.04-1.61) | 1.30 (1.04-1.59) | 1.24 (1.03-1.63) | 0.521 |
| LDL-C, mmol/L | 3.72 (2.82-4.95) | 3.84 (3.04-5.17) | 3.67 (2.64-4.82) | 0.082 |
| TG, mmol/L | 2.28 (1.45-3.62) | 2.32 (1.60-3.92) | 2.22 (1.43-3.59) | 0.391 |

^*^: *P* < 0.05; ^**^: *P* < 0.01.

Supple. Table 2. Baseline characteristics of 495 patients according to glomerular PLA2R deposit.

| Characteristics | All patients  (N=495) | PLA2R deposit (+)  (N=406) | PLA2R deposit (-)  (N=89) | *P*-value |
| --- | --- | --- | --- | --- |
| Demographics |  |  |  |  |
| Age, y | 49 (39- 56) | 49 (41-57) | 44 (31-54) | 0.004**^**^** |
| Male sex, no. (%) | 287 (57.9) | 252 (59.1) | 35 (39.3) | < 0.001**^**^** |
| Anthropometric Measurements |  |  |  |  |
| Systolic BP, mmHg | 127 (118-139) | 127 (118-139) | 127 (115-138) | 0.366 |
| Diastolic BP, mmHg | 82 (75-91) | 83 (75-91) | 80 (72-91) | 0.060 |
| BMI, kg/m^2^ | 24.1 (22.1-26.4) | 24.2 (22.3-26.5) | 23.0 (20.8-25.6) | 0.006**^**^** |
| Kidney Function Measurements |  |  |  |  |
| Serum creatinine, μmol/L | 75 (60-90) | 76 (62-92) | 69 (52-88) | 0.014**^*^** |
| Urine protein, g/d | 3.8 (1.8-6.0) | 4.1 (2.1-6.4) | 2.6 (0.9-5.4) | < 0.001**^**^** |
| Albumin, g/L | 28.8 (23.5-34.0) | 28.2 (23.2-32.7) | 30.5 (23.7-38.2) | 0.028**^*^** |
| Plasma Lipid Levels |  |  |  |  |
| TC, mmol/L | 6.38 (5.14-7.88) | 6.62 (5.37-8.04) | 5.45 (4.21-7.23) | < 0.001**^**^** |
| Non-HDL-C, mmol/L | 4.96 (3.85-6.51) | 5.12 (4.07-6.73) | 4.01 (3.05-5.57) | < 0.001**^**^** |
| HDL-C, mmol/L | 1.27 (1.04-1.61) | 1.29 (1.05-1.61) | 1.21 (0.91-1.61) | 0.067 |
| LDL-C, mmol/L | 3.72 (2.82-4.95) | 3.86 (2.92-5.01) | 3.18 (2.33-4.60) | 0.002**^**^** |
| TG, mmol/L | 2.28 (1.45-3.62) | 2.31 (1.53-3.86) | 2.06 (1.19-3.03) | 0.023**^*^** |

^*^: *P* < 0.05; ^**^: *P* < 0.01.

Supple. Table 3. Baseline characteristics of 124 patients according to seropositivity of anti- PLA2R antibody.

| Characteristics | All patients  (N=124) | Anti-PLA2R antibody (+)  (N=70) | Anti-PLA2R antibody (-)  (N=54) | *P*-value |
| --- | --- | --- | --- | --- |
| Demographics |  |  |  |  |
| Age, y | 51 (41-56) | 52 (44-56) | 47 (32-55) | 0.108 |
| Male sex, no. (%) | 77 (62.1) | 47 (67.1) | 30 (55.5) | 0.187 |
| Anthropometric Measurements |  |  |  |  |
| Systolic BP, mmHg | 126 (117-139) | 127 (117-138) | 126 (115-139) | 0.256 |
| Diastolic BP, mmHg | 83 (76-92) | 84 (76-91) | 81 (73-93) | 0.359 |
| BMI, kg/m^2^ | 24.4 (22.2-26.2) | 24.6 (22.9-27.1) | 23.2 (21.6-25.8) | 0.045**^*^** |
| Kidney Function Measurements |  |  |  |  |
| Serum creatinine, μmol/L | 73 (61-90) | 83 (64-99) | 71 (54-82) | 0.021**^*^** |
| Urine protein, g/d | 3.5 (1.8-6.0) | 4.8 (2.7-7.8) | 2.1 (1.0-3.3) | < 0.001**^**^** |
| Albumin, g/L | 29.6 (24.5-35.4) | 26.8 (23.2-31.2) | 32.6 (27.7-37.5) | < 0.001**^**^** |
| Plasma Lipid Levels |  |  |  |  |
| TC, mmol/L | 6.18 (5.04-7.87) | 6.55 (5.42-8.46) | 5.66 (4.25-7.06) | 0.005**^**^** |
| Non-HDL-C, mmol/L | 4.70 (3.57-6.64) | 5.11 (4.05-7.13) | 4.05 (3.11-5.64) | 0.001**^**^** |
| HDL-C, mmol/L | 1.34 (1.03-1.67) | 1.27 (1.04-1.62) | 1.39 (1.00-1.79) | 0.602 |
| LDL-C, mmol/L | 3.73 (2.65-4.83) | 4.21 (3.03-5.55) | 3.11 (2.20-4.05) | < 0.001**^**^** |
| TG, mmol/L | 2.17 (1.52-3.66) | 2.29 (1.66-3.56) | 2.09 (1.34-4.29) | 0.689 |

^*^: *P* < 0.05; ^**^: *P* < 0.01.

Supple. Table 4. Characteristics of 236 patients after treatment according to proteinuria outcomes.

| Characteristics | All patients  (N=236) | Without remission  (N=95) | Remission  (N=141) | *P*-value |
| --- | --- | --- | --- | --- |
| Demographics |  |  |  |  |
| Age, y | 49 (41-57) | 50 (43-57) | 49 (39-56) | 0.305 |
| Male sex, no. (%) | 145 (61.4) | 62 (65.3) | 83 (58.8) | 0.229 |
| Anthropometric Measurements |  |  |  |  |
| Systolic BP, mmHg | 129 (118-140) | 130 (118-140) | 129 (118-140) | 0.858 |
| Diastolic BP, mmHg | 82 (75-89) | 84 (75-91) | 80 (76-87) | 0.113 |
| BMI, kg/m^2^ | 24.3 (22.4-27.1) | 24.4 (21.9-26.9) | 24.3 (22.5-27.2) | 0.821 |
| Kidney Function Measurements |  |  |  |  |
| Serum creatinine pre-treatment, μmol/L | 79 (63-98) | 83 (65-100) | 77 (63-96) | 0.470 |
| Serum creatinine post-treatment, μmol/L | 78 (64-92) | 85 (70-106) | 73 (60-88) | < 0.001**^**^** |
| Urine protein pre-treatment, g/d | 4.3 (2.4-7.4) | 3.9 (2.4-6.2) | 4.5 (2.6-7.6) | 0.153 |
| Urine protein post-treatment, g/d | 1.6 (0.4-3.8) | 4.8 (2.7-7.9) | 0.5 (0.2-1.4) | < 0.001**^**^** |
| Albumin pre-treatment, g/L | 26.8 (22.8-32.6) | 26.7 (22.1-30.5) | 27.9 (24.2-34.1) | 0.026**^*^** |
| Albumin post-treatment, g/L | 33.3 (27.2-38.0) | 26.6 (21.6-31.6) | 36.1 (32.9-39.9) | < 0.001**^**^** |
| Plasma Lipid Levels post-treatment |  |  |  |  |
| TC, mmol/L | 5.75 (4.82-7.21) | 6.63 (5.56-8.98) | 5.40 (4.45-6.24) | < 0.001**^**^** |
| Non-HDL-C, mmol/L | 4.26 (3.30-5.70) | 5.21 (3.92-7.12) | 3.84 (3.03-4.77) | < 0.001**^**^** |
| HDL-C, mmol/L | 1.52 (1.20-1.98) | 1.32 (1.18-1.87) | 1.87 (1.20-2.01) | 0.006**^**^** |
| LDL-C, mmol/L | 3.48 (2.61-4.46) | 4.11 (3.06-5.42) | 3.29 (2.42-3.96) | < 0.001**^**^** |
| TG, mmol/L | 1.90 (1.22-2.87) | 2.31 (1.39-3.25) | 1.65 (1.16-2.43) | 0.004**^**^** |

^*^: *P* < 0.05; ^**^: *P* < 0.01.
